# 18β-glycyrrhetinic acid suppresses gastric cancer by activation of miR-149-3p-Wnt-1 signaling

**DOI:** 10.18632/oncotarget.12443

**Published:** 2016-10-04

**Authors:** Donghui Cao, Zhifang Jia, Lili You, Yanhua Wu, Zhen Hou, Yueer Suo, Houjun Zhang, Simin Wen, Tetsuya Tsukamoto, Masanobu Oshima, Jing Jiang, Xueyuan Cao

**Affiliations:** ^1^ Division of Clinical Research, First Hospital of Jilin University, Changchun, Jilin 130021, China; ^2^ Department of Gastric and Colorectal Surgery, First Hospital of Jilin University, Changchun, Jilin 130021, China; ^3^ Department of Diagnostic Pathology I, School of Medicine, Fujita Health University, Toyoake, 470-1192, Japan; ^4^ Cancer Research Institute, Kanazawa University, Kanazawa, 920-1192, Japan

**Keywords:** 18β-glycyrrhetinic acid, gastric cancer, COX-2, miR-149-3p, Wnt-1

## Abstract

18β-glycyrrhetinic acid (GRA) exerts anti-tumor effects on various types of cancer. In the present study, we found that GRA attenuated the severity of gastritis and suppressed gastric tumorigenesis in transgenic mice. We also discovered that miR-149-3p was downregulated in gastric cancer tissues and cell lines as compared to normal gastric tissues and epithelial cells, but was upregulated by GRA. miR-149-3p expression also correlated negatively with lymphnode metastasis. Our functional assays showed that miR-149-3p overexpression inhibited cell proliferation and cell cycle progression while inducing apoptosis, while inhibition of miR-149-3p had the opposite effects. In addition, we identified Wnt-1 as a direct target of miR-149-3p. These data suggest that GRA inhibits the initiation and progression of gastric tumors by ameliorating the inflammatory microenvironment through downregulation of COX-2 expression and by inhibiting Wnt-1 expression through the upregulation of tumor suppressor miR-149-3p. GRA may thus have the potential to serve as a useful therapeutic agent for the prevention and treatment of gastric cancer.

## INTRODUCTION

Gastric cancer (GC) is the fifth most common malignancy (915,600 cases) and the third cause of cancer-related deaths (723,100 cases) worldwide [1]. More specifically, GC ranks 2nd in cancer incidence (424,000 cases) and 3rd (298,000 cases) in cancer mortality in China [2]. Most GC patients are diagnosed at an advanced-stage of the disease and given a poor prognosis. The main treatment consists in gastrectomy combined with chemotherapy and radiotherapy; however, recurrence and metastasis are common. This has prompted the rapid development of alternative targeted approaches, such as immunotherapy and treatment with antibodies or phytochemicals [3–5].

While gastric cancer is a multifactorial disease, inflammation is one of the main drivers of tumorigenesis [6]. The most common histologic variant of gastric adenocarcinoma is the intestinal-type, and its typical cascade model consists of chronic superficial (non-atrophic) gastritis, chronic atrophic gastritis, intestinal metaplasia, dysplasia and finally gastric adenocarcinoma [7]. The COX-2/PGE2 pathway acts as a tumor promoter during the generation of the inflammatory microenvironment. The transgenic mouse model K19-C2mE, engineered by the Oshima group [8], overexpressed *Cox-2* and *mPGES-1* in gastric mucosa epithelial cells, resulting in the development of hyperplasia, metaplasia and tumorigenesis in the proximal end of the gastric mucosa. In our previous study [9], Canolol decreased the inflammatory response in gastric mucosa epithelia, and inhibited gastric tumorigenesis by blocking the COX-2/PGE2 pathway.

Inflammation promotes tumorigenesis by enhancing ROS production, thereby causing silent mutations in oncogenes, and by activation of the well-known NF-κB and MAPK pathways [10]. Accumulation of β-catenin, a hallmark of Wnt signaling activation, is found in more than 50% of gastric cancers [11]. Several drugs such as pantoprazole [12] and salinomycin [13], and some natural products such as γ-tocotrienol, *Capsosiphon fulvescens* glycoprotein (Cf-GP), diphyllin, and flavanone, can prevent GC cell proliferation and invasiveness by targeting the Wnt/β-catenin signaling pathway [14–17].

The major bioactive component in licorice root, 18β-glycyrrhetinic acid (GRA), exerts anti-inflammatory, anti-oxidative and anti-cancer effects [18–20]. Indeed, GRA is contained in the compound Hongdoushan capsule (CHC), used to treat ovarian and breast cancers [21]. Furthermore, previous studies revealed that GRA can act as a tumor suppressor in breast [19], lung [22] and gastric [23] cancers. Additionally, GRA significantly reduced cisplatin-induced nephrotoxicity through the Nrf2/NF-kappaB signaling pathway in BALB/c mice [24]. In our previous study [18], GRA attenuated *H. pylori*-infected gastritis in gerbils; however, further studies are needed to elucidate the mechanisms by which GRA inhibits tumorigenesis *in vivo* and *in vitro*.

miRNAs are a class of small noncoding RNAs (18–25 nucleotides in length) that regulate gene expression post-transcriptionally. miRNAs play either a tumor-suppressive or an oncogenic role in cancer by regulating their target genes. Expression profiling has shown that genes in the COX-2/PGE-2 and Wnt/β-catenin pathway are affected by dysregulation of miRNAs in gastric cancer [25]. For example, miR-7 suppressed active chronic gastritis and gastric cancer by targeting *Cox-2* [26] and miR-1225-5p constrained GC growth and metastatic potential via inhibition of β-catenin signaling [27]. In the present study, we hypothesized that miRNAs might be involved in the inhibitory effects of GRA on gastric carcinogenesis. We demonstrated that GRA changed miRNA expression profiles, improved the inflammatory microenvironment, inhibited gastric tumorigenesis in Tg mice, and suppressed cell proliferation *in vitro*. We also showed that GRA treatment upregulated miR-149-3p expression, which inhibited cell cycle progression and induced cell apoptosis in gastric tumor cells by suppressing the expression of its direct target gene *Wnt-1*. Our study suggests that GRA could be exploited as a chemoprophylactic and therapeutic agent against gastric cancer.

## RESULTS

### GRA suppressed chronic active gastritis and downregulated the inflammation-related genes in K19-C2mE Tg mice

**Figure 1:**
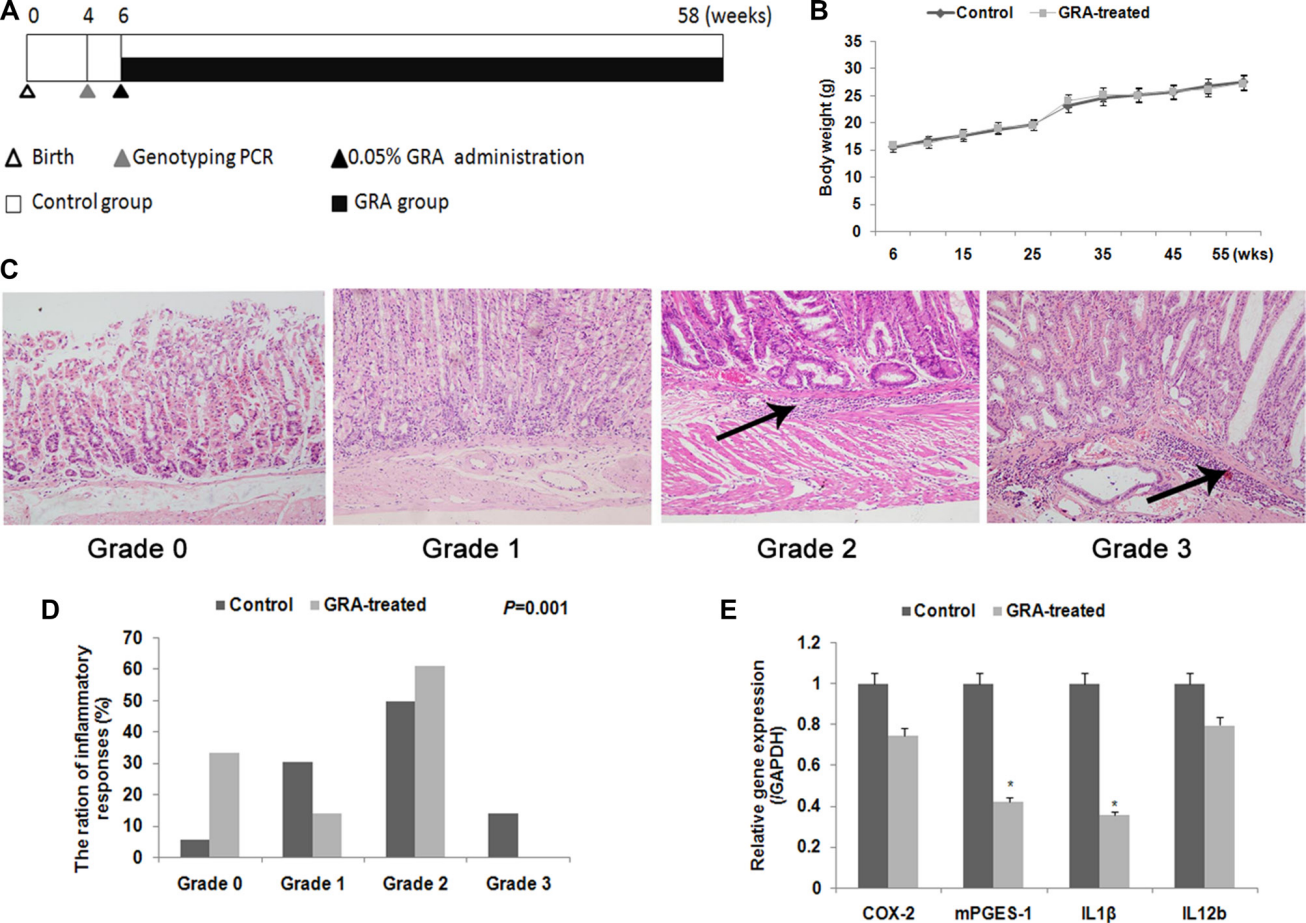
GRA treatment prevents gastritis and inflammatory cytokine production in transgenic mice (**A**) Experimental design in this study. (**B**) Body weight of mice during the GRA treatment period. No significant difference was noted. (**C**) Histologic grades in Tg mice in the two groups. Arrows showed the infiltration of neutrophils, lymphocytes, and macrophages into the submucosa. (**D**) The percentage and quantification indicates the histopathology changes in Tg mice in the two groups. (**E**) Various inflammatory cytokines were decreased after GRA treatment. *P < 0.05.

The transgenic mice were divided into control (*n* = 40) and 0.05% GRA-treated groups (*n* = 40) randomly after genotyping (Figure 1A). At the end of the animal experiments, the survival rates were 90% (36/40) for the control group and 97.5% (39/40) for the GRA-treated group. GRA administration was well tolerated in Tg mice, and there were no significant differences in body weight or growth curves (Figure 1B).

We first conducted histopathological experiments to test for the effects of GRA on gastritis intransgenic mice. A four-point scale (G0, normal; G1, mild; G2, moderate; and G3, marked gastritis) was used to grade chronic active gastritis. HE staining showed active inflammatory mucosal changes, with many neutrophils, lymphocytes, and macrophages heavily infiltrating the gastric submucosa in the control group (Figure 1C). On the contrary, infiltration was reduced in the GRA-treated group (*P* = 0.001, Figure 1D). Then we assayed the mRNA levels of inflammatory cytokines, *Cox-2*, *mPGES-1*, *Il-1*β, and *Il-12b* by qRT-PCR. The results showed that GRA administration downregulated the expression of *mPGES-1* to 41.95% and *Il-1β* to 35.41% (Figure 1E).

### GRA markedly reduced the tumor incidence and development *in vivo*

**Figure 2:**
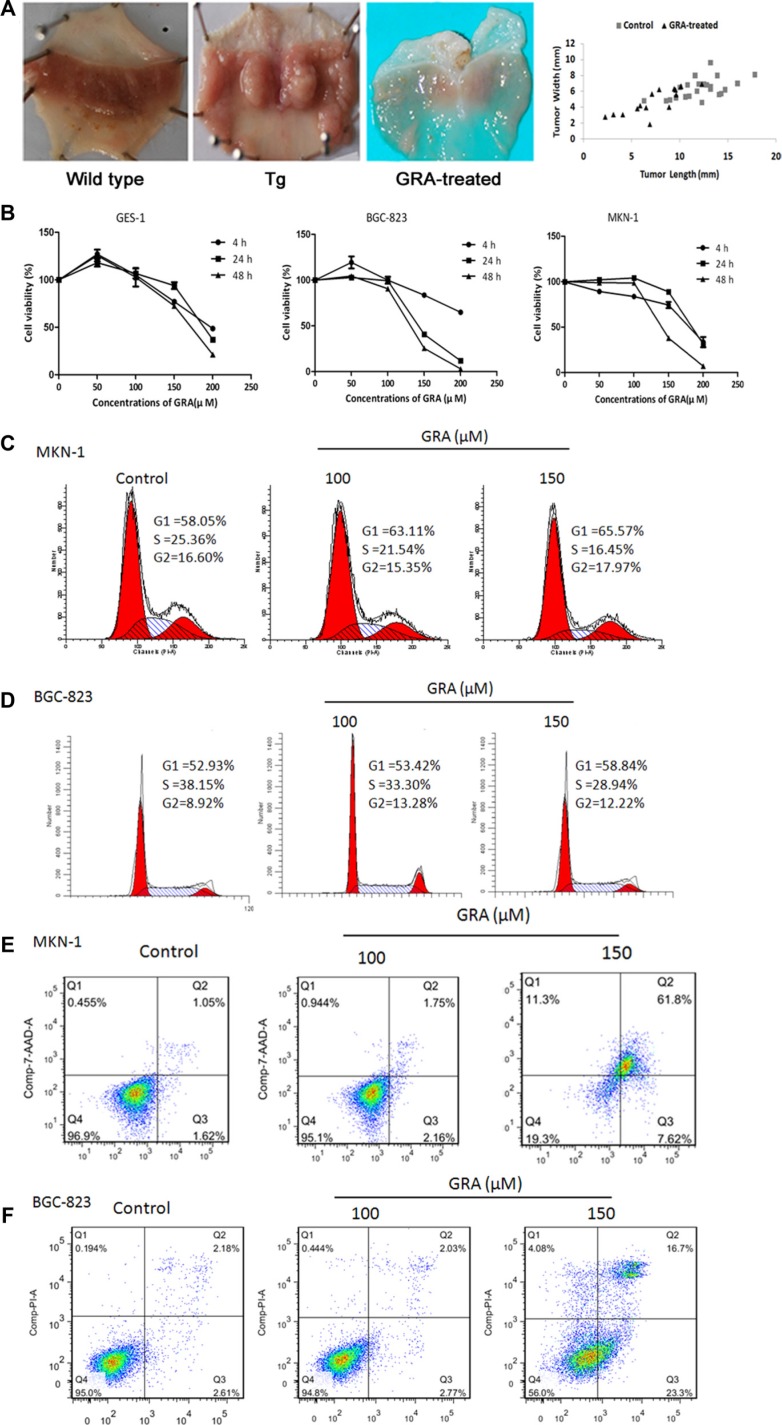
GRA treatment inhibited the development and progression of gastric tumor *in vivo*, and suppressed cell proliferation and cell cycle progression *in vitro* (**A**) Macroscopic appearance of the stomach of Tg mice in control and GRA-treated groups, compared with wild type mice (left). Representative quantification (right) of the tumor length and width in the GRA-treated and control groups (mean ± SD). (**B**) GRA decreased the viability of the tumor cell lines BGC-823 and MKN-1 in a time- and dose-dependent manner as measured by the MTT assay, and the normal mucosa cell line GES-1 was used as control. GRA induced G0/G1 phase arrest and enhanced apoptosis in (**C**–**E**). MKN-1 and (**D–F**) BGC-823 cells after exposure for 24 h.

The effects of GRA on GC development and progression were examined in Tg mice. The results showed that, in the control group, the incidence of gastric tumor was 77.8% (28/36), the mean length of tumor tissue was 12.1 ± 2.5 mm, and the mean width was 6.3 ± 1.3 mm. In contrast, in the GRA-treated group, the tumor incidence was 33.4% (13/39), the mean length was 7.4 ± 2.8 mm, and the mean width was 4.8 ± 1.6 mm. The results suggested that GRA suppressed the incidence (*P* = 0.002), length (*P* < 0.001) and width (*P* = 0.002) of gastric tumors (Table 1, Figure 2A).

**Table 1:** The tumor incidence in the control group and GRA-treated group

Group	Mice-bearing tumors		Mice without tumors		*P* value
	Number	Incidence	Number	Incidence	
Control (*n* = 36)	28	77.8%	8	22.2%	0.002
GRA-treated (*n* = 39)	13	33.4%	26	66.6%	

### GRA suppressed tumor cell viability and induced cell apoptosis *in vitro*

Considering the intestinal and liver metabolism of Tg mice, the gradient concentrations of GRA (50–300 μM) were designed in the preliminary experiment *in vitro*. Then, serial concentrations of GRA, 50 μM, 100 μM, 150 μM, 200 μM were adopted to treat gastric tumor cells BGC-823 and MKN-1 for 4 h, 24 h and 48 h. The human immortal gastric epithelial cell line GES-1 was used as control.

MTT assays showed that tumor cell proliferation was slightly increased by 50 μM GRA in BGC-823 (119.30%) and GES-1 (127.00%) cells for 4 h. On the other hand, proliferation was not influenced by 100 μM GRA in BGC-823, MKN-1 and GES-1 cells after 4 h and 24 h. However, when the GRA concentration reached to 150 μM, cell proliferation was inhibited in BGC-823 for 24 h (40.70%) and 48 h (25.44%), while GES-1 proliferation was 93.95% for 24 h and 27.47% for 48 h. Furthermore, MTT results also showed that 200 μM GRA had measurable effects on cell viability. BGC-823 and MKN-1 proliferation was decreased to 64.76% and 33.59% independently. GES-1 proliferation was also decreased to 48.73% for 4 h. These results indicated that GRA inhibited gastric tumor cell proliferation in a dose- and time-dependent manner, and that 150 μM was the ideal concentration causing less side-effects in normal epithelial cells (Figure 2B).

Flow cytometry analyses of the cell cycle showed that the number of cells at G0/G1 phase was 58.05% for MKN-1, and 52.93% for BGC-823. After exposure to 150 μM GRA for 24 h, the accumulation of cells reached a peak value of 65.57% for MKN-1 (Figure 2C), and 58.84% for BGC-823 (Figure 2E). Meanwhile, the number of cells in S phase was 25.36% for MKN-1, and 38.15% for BGC-823, while they were decreased to 16.45% in MKN-1, and 28.94% in BGC-823. The results showed that GRA inhibited cell cycle progression independently from the differentiation status of the cells.

In addition, flow cytometry analyses also showed that the apoptosis rate was 1.62% in MKN-1, increasing to 7.62% after 150 μM GRA treatment for 24 h (Figure 2D), and it was 2.61% in BGC-823, increasing to 23.30% after GRA treatment for 24 h (Figure 2F).

### COX-2 and canonical Wnt pathway were the possible targets of GRA

**Figure 3:**
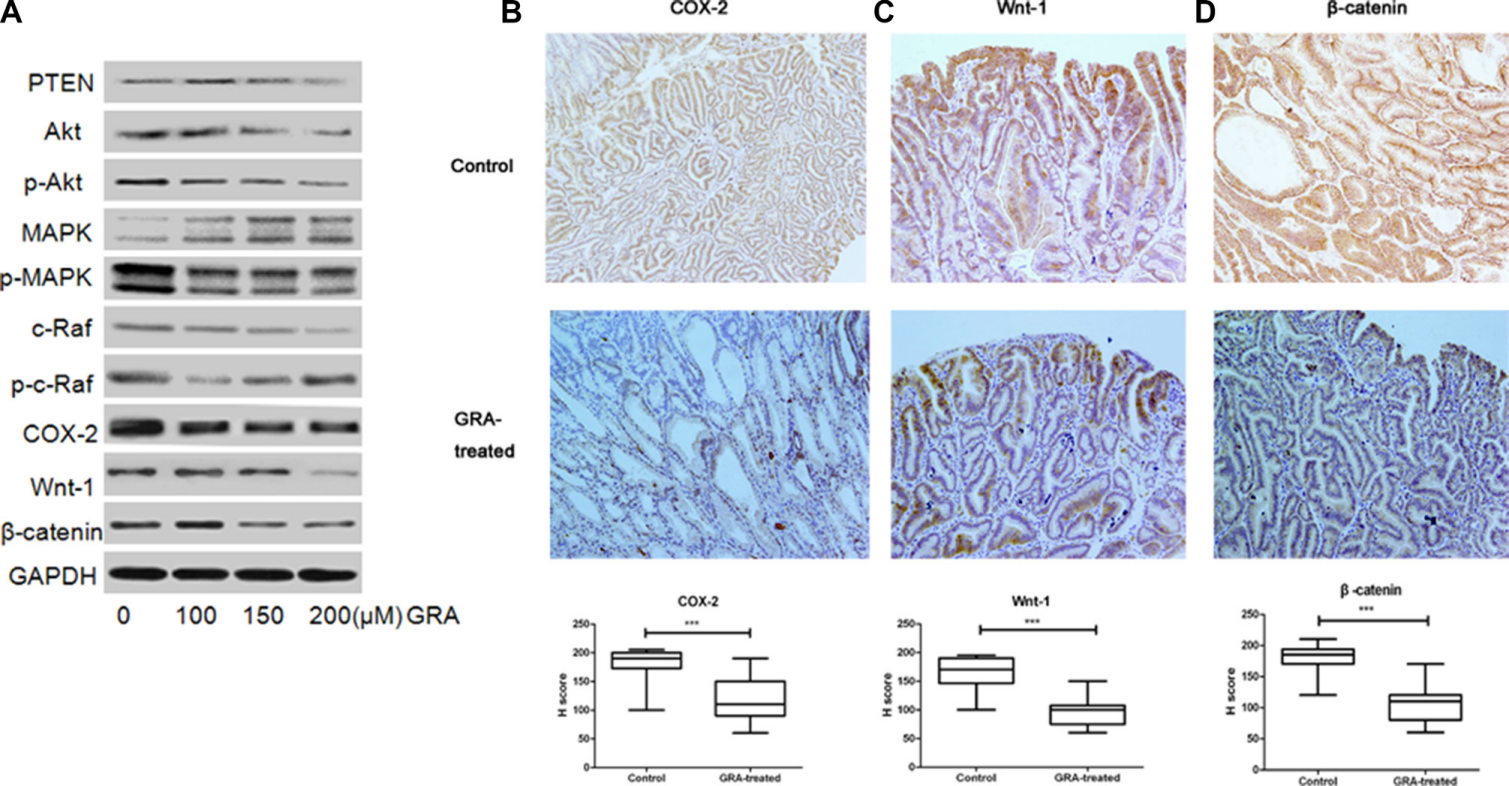
GRA decreased the expression of canonical Wnt pathway genes (**A**) The effects of GRA on the levels of several key genes, PTEN, Akt, p-Akt (Ser 473), p44/42 MAPK (Erk1/2) and p-p44/42 MAPK (Erk1/2) (Thr202/Tyr204), c-Raf, p-c-Raf (Ser 338), COX-2, Wnt-1, β-catenin. The IHC staining and H score of (**B**) COX-2, (**C**) Wnt-1 and (**D**). β-catenin in the stomach of Tg mice treated with GRA, compared with the control mice (magnification × 20).

To explore the possible targets of GRA, scanning experiments considering COX-2/PGE2, PI3K/Akt and MAPK/ERK signaling pathways were conducted. COX-2, Wnt-1, β-catenin, PTEN, Akt, p-Akt(Ser 473), c-Raf, p-c-Raf (Ser 338), p44/42 MAPK (Erk1/2) and p-p44/42 MAPK (Erk1/2) (Thr202/Tyr204) were chosen as candidates of these pathways. Western blot showed that PTEN, Akt, p-Akt, c-Raf expressions were slightly decreased; however, expressions of COX-2, Wnt-1, β-catenin, and p-MAPK were decreased markedly after GRA administration (Figure 3A).

To further investigate the expression locations of COX-2, Wnt-1 and β-catenin, we analyzed immunohistochemically stained gastric tumor tissue. Immunostaining of COX-2 was observed mainly in the membrane and cytoplasm of gastric mucosa cells in the control group, while it was predominantly located on the cell membrane in the GRA-treated group (Figure 3B). β-catenin accumulated mainly in the cell cytoplasm and nucleus in the control group; however, the accumulation was much lighter in cytoplasm and absent in the nucleus after GRA treatment (Figure 3C). Similarly, Wnt-1, was mainly stained in the cell membrane and cytoplasm in the control group, and it was predominantly stained in the cytoplasm after GRA administration (Figure 3D). Furthermore, the H score indicated that the expression of COX-2, Wnt-1 and β-catenin was downregulated significantly by GRA administration (*P* < 0.001).

All the results of IHC, qRT-PCR and western blot indicated that COX-2 and the canonical Wnt pathway were the possible targets of GRA.

### miR-149-3p was upregulated by GRA administration in a dose- and time-dependent manner

**Figure 4:**
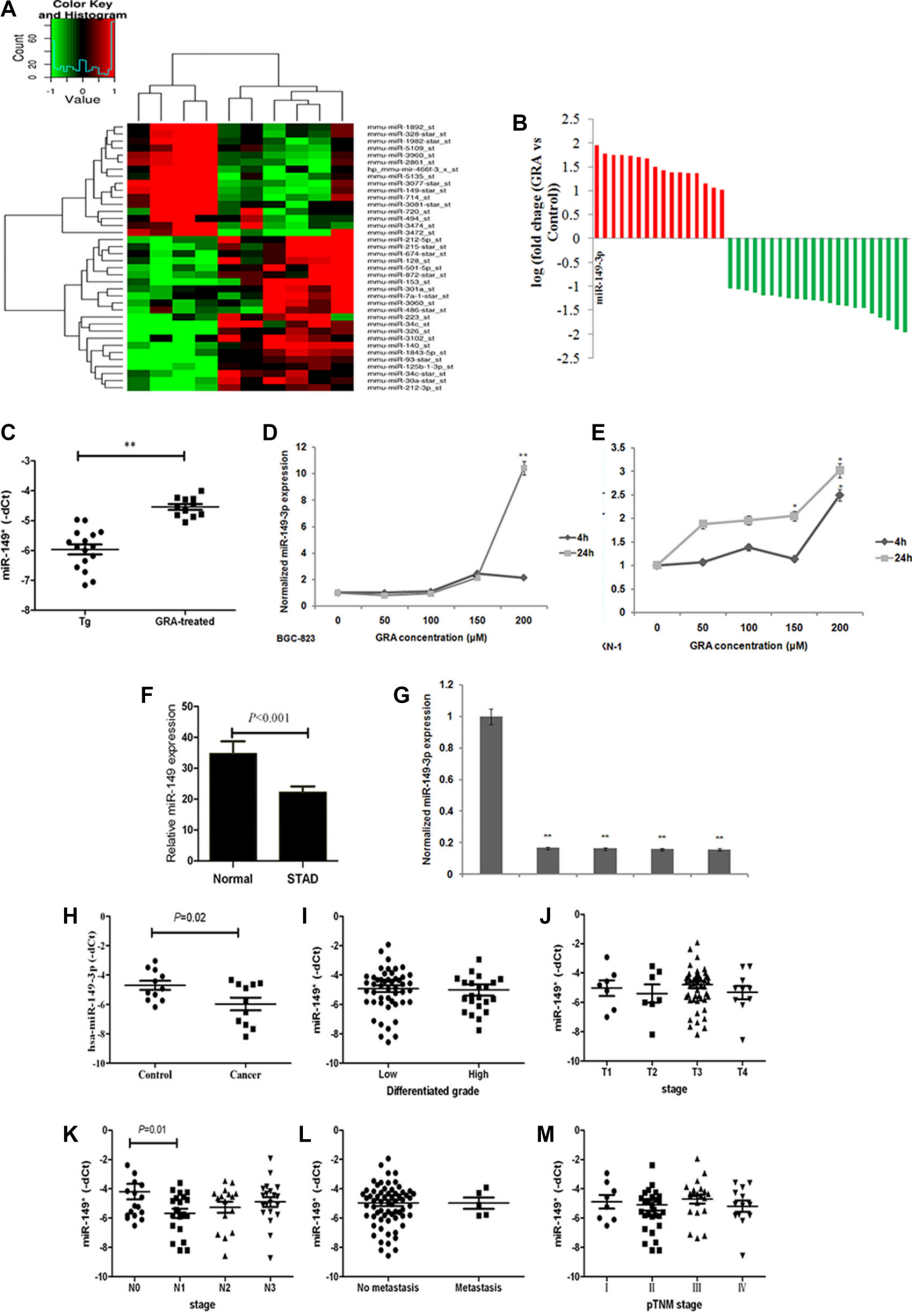
miR-149-3p was upregulated after GRA administration, and frequently downregulated in GC tissues and cells (**A**) MiRNA expression profile revealed upregulated and downregulated miRNAs in the GRA-treated group compared to the control group. (**B**) miR-149-3p was upregulated the most by GRA. (**C**) miR-149-3p upregulation was verified in enlarged samples of Tg mice. GRA upregulated miR-149-3p expression in (**D**) BGC-823 and (**E**) MKN-1 in a time- and dose-dependent manner. (**F**) miR-149-3p expression in STAD compared with normal tissue deposited in the TCGA database. (**G**) miR-149-3p expression in gastric tumor cells compared with normal mucosa cells. (**H**) miR-149-3p expression in gastric tumor tissues compared with paired normal epithelia mucosa. The correlation analysis of miR-149-3p expression with (**I**) differentiated grade of tumor, (**J**) T stage, (**K**) N stage, (**L**) M stage, and (**M**) pTNM stages.

To examine whether miRNA expression is regulated after GRA administration, the miRNA profiles in the control group (*n* = 5) and GRA-treated group (*n* = 5) were examined (Figure 4A). Overall, the transcriptional levels of 38 miRNAs were altered, 22 miRNAs were upregulated (fold change ≥ 2, *P* < 0.05), and 16 miRNAs were downregulated (fold change ≤ 0.5, *P* < 0.05) in the GRA-treated group (Supplementary Table S1). Among these miRNAs, miR-149-3p was upregulated the most by 3.84-fold (*P* < 0.001) (Figure 4B). In addition, more gastric tumor tissues were used to confirm the results of the microarray analysis, and qRT-PCR showed that −ΔCt in the control group (*n* = 16) was 5.96, and 4.54 in GRA-treated group (*n* = 11) (Figure 4C), the results pointed out that GRA indeed upregulated the expression of miR-149-3p.

The miR-149-3p sequence is highly conserved in *Mus musculus* and *Homo sapiens*. Thus, gastric tumor cells BGC-823 and MKN-1 were chosen to detect the effects of GRA on the expressions of miR-149-3p, and qRT-PCR results demonstrated that miR-149-3p levels were upregulated by GRA in a time- and dose-dependent manner (Figure 4D and 4E).

### miR-149-3p was frequently downregulated in human gastric cancer and negatively associated with the presence of lymph node metastasis

To further ascertain miR-149-3p expression in human gastric cancer, stomach adenocarcinoma (STAD) and normal samples deposited in the TCGA database (*n* = 432) were analyzed. The miR-149-3p level was 34.74 ± 25.70 in normal tissue (*n* = 42) and 22.19 ± 38.05 in STAD (*n* = 390) (Figure 4D). Additionally, miR-149-3p was highly expressed in the normal epithelial cell line GES-1, while its levels were reduced in various gastric tumor cell lines, such as AGS, BGC-823, MGC-803 and MKN-1 (*P* < 0.001, Figure 4E).

To address the clinical impact of miR-149-3p expression in gastric cancer, we used qRT-PCR to analyze 11 cases of GC tissues and paired control tissues collected in our hospital. Compared with control tissues, the median level of miR-149-3p was lower in the gastric tumor tissue (*P* = 0.02, Figure 4H), consistent with TCGA data. In addition, to explore the relationship between miR-149-3p expression and GC progression, 74 cases of gastric cancer with intact clinical pathological information were divided into two groups according the median level of miR-149-3p expression. Results from our analyses showed that low levels of miR-149-3p correlated positively with the presence of lymphonode metastasis (*P* = 0.01, Figure 4K). However, we detected no correlation with tumor differentiation (Figure 4I), depth of invasion (Figure 4J), distant metastasis (Figure 4L) and pTNM stage (Figure 4M).

### miR-149-3p suppressed GC cell proliferation and cell cycle progression *in vitro*

**Figure 5:**
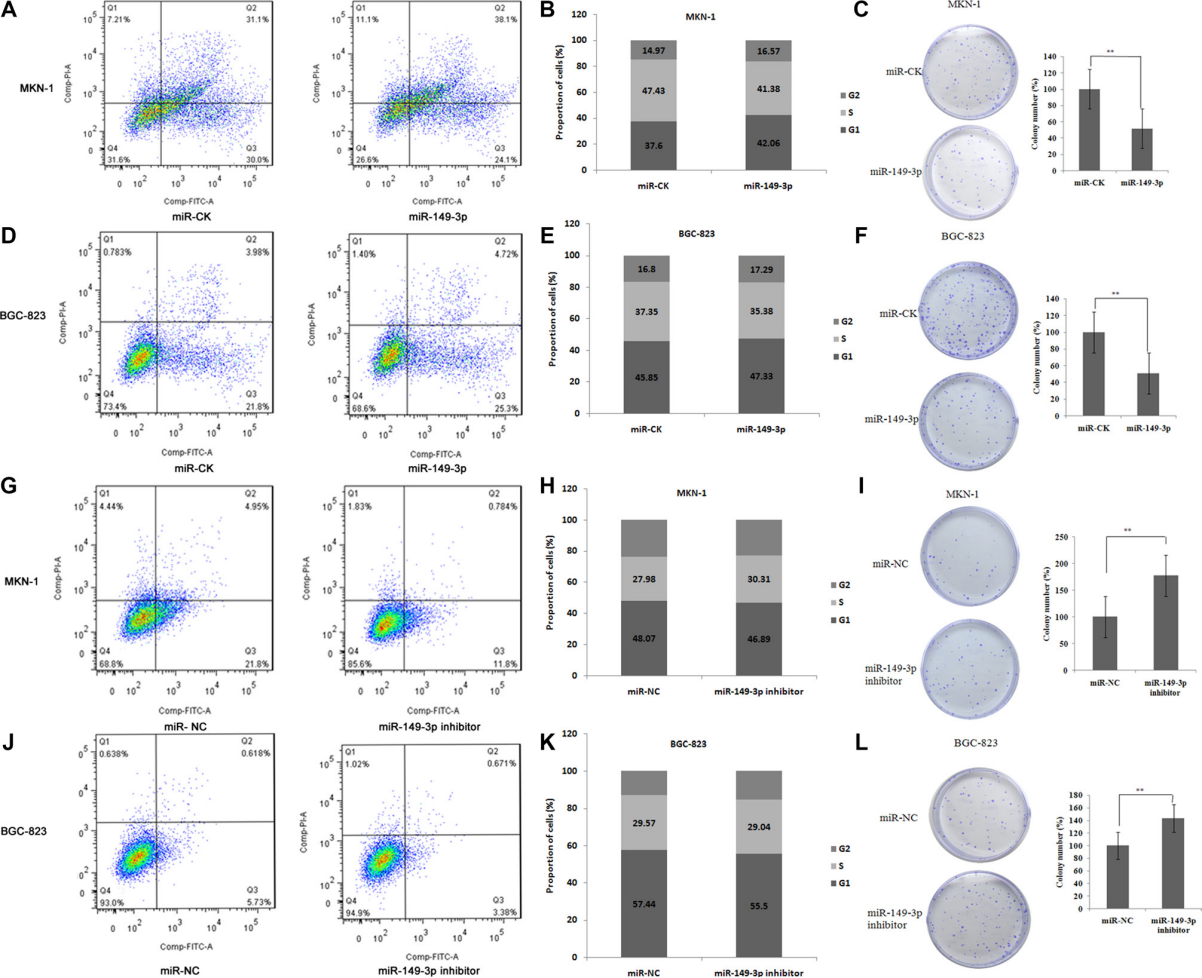
miR-149-3p induced cell apoptosis and suppressed cell cycle progression Overexpression of miR-149-3p induced (**A**) cell apoptosis, (**B**) G0/G1 arrest, and (**C**) suppressed cell colony formation in MKN-1 cells. Overexpression of miR-149-3p induced (**D**) cell apoptosis, (**E**) G0/G1 arrest, and (**F**) suppressed cell colony formation in BGC-823 cells. Inhibition of miR-149-3p suppressed (**G**) cell apoptosis, (**H**) G0/G1 arrest, and (**I**) stimulated cell colony formation in MKN-1 cells. Inhibition of miR-149-3p suppressed (**J**) cell apoptosis, (**K**) G0/G1 arrest, and (**L**) stimulated cell colony formation in BGC-823 cells.

Next, we used MKN-1 and BGC-823 cells to monitor the impact of ectopic overexpression of miR-149-3p on cell cycle, apoptosis and reproducibility. Results from our analyses showed that miR-149-3p overexpression induced apoptosis, increased the numbers of cells in G0/G1 phase and G2/M phase while decreasing those in S phase, and led to smaller and fewer colonies compared to miR-CK cells (*P* < 0.01) both in MKN-1 (Figure 5A–5C) and BGC-823 (Figure 5D–5F).

In contrast, transfection with miR-149-3p inhibitors led to decreased apoptosis compared to the miR-NC group, fewer cells in G0/G1 and G2/M arrest, and higher cell reproducibility (*P* < 0.01) in both MKN-1 (Figure 5G–5I) and BGC-823 (Figure 5J–5L) cells.

### Wnt-1 is a direct target gene of miR-149-3p

**Figure 6:**
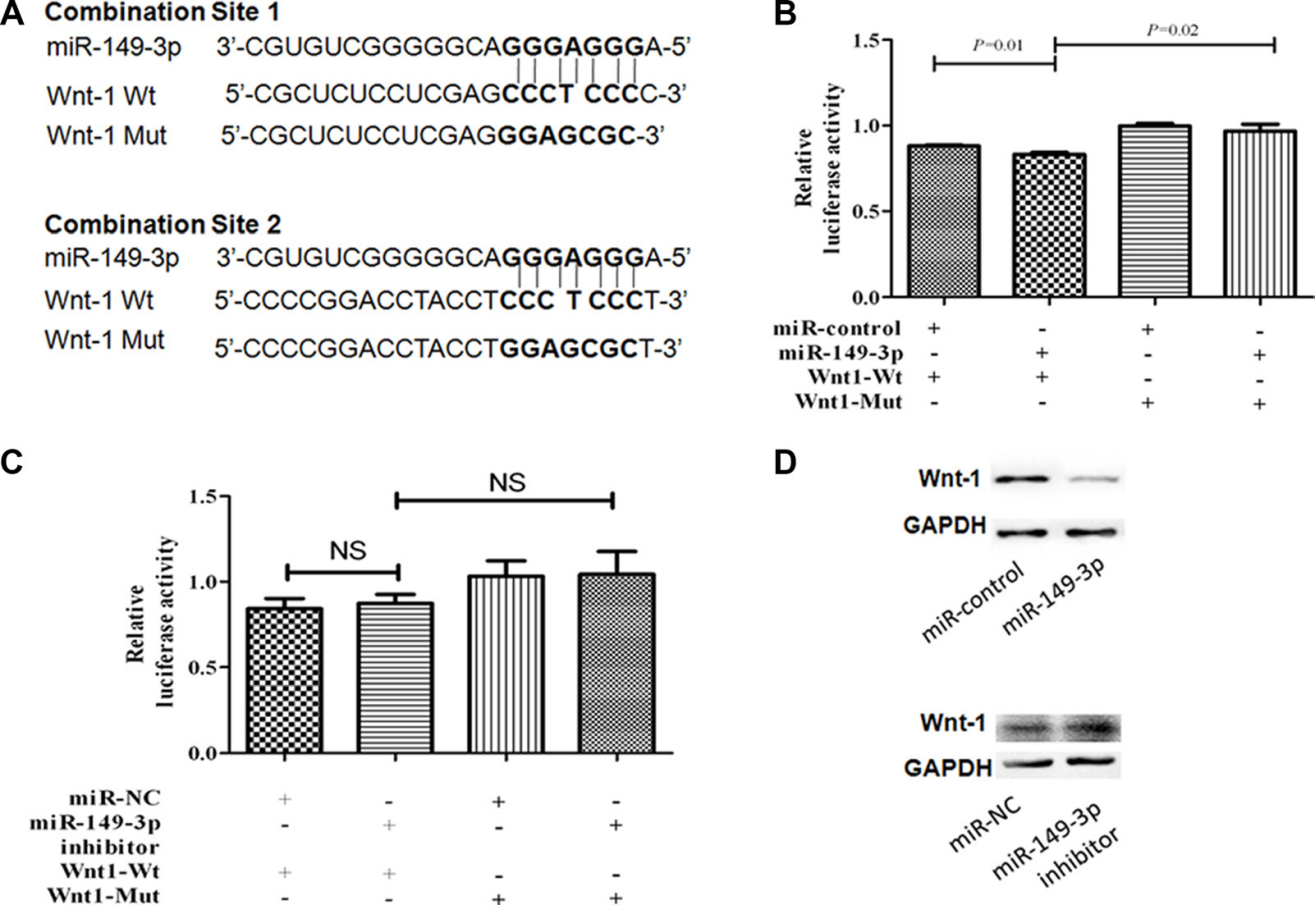
MiR-149-3p directly targets the 3′-UTR of *Wnt-1* mRNA (**A**) Schematic representation of the mature miR-149-3p sequence, the target sequence in the 3′-UTR of *Wnt-1*, and a 3′-UTR mutant of *Wnt-1* containing seven mutated nucleotides in the putative target site. (**B**) HEK-293T cells were cotransfected with pGL3-miR-149-3p plasmid or miR-CK and pmiRGLO-Wnt-1-WT or pmiRGLO-Wnt-1-Mut reporter plasmid for 24 h. (**C**) HEK-293T cells were cotransfected with miR-149-3p inhibitor or miR-NC and pmiRGLO-Wnt-1-WT or pmiRGLO-Wnt-1-Mut reporter plasmid for 24 h. Each bar represents the mean ± SE of three independent experiments. (**D**) Western blot of Wnt-1 expression after transfection with pGL3-miR-149-3p plasmid or miR-149-3p inhibitor. **P < 0.01.

To find potential target genes of miR-149-3p in the COX-2 and the canonical Wnt pathways, we used miRDB software (http://www.mirdb.org/miRDB/) and the KEGG pathway database (http://www.genome.jp/kegg/pathway.html). *Wnt-1* was predicted to be an important target gene of miR-149-3p with two complementary sites in its 3′-UTR using the online software TargetScan (http://www.targetscan.org/) and miRanda-mirSVR (http://www.microrna.org/) (Figure 6A). Hereby, we constructed pmirGLO vectors containing the binding sites of miR-149-3p in 3′-UTR of *Wnt-1* and pGL3 vector containing miR-149-3p sequence independently. The pGL3-miR-149-3p and pmirGLO-Wnt1-WT vectors were cotransfected into HEK293T cells, and luciferase assays showed that overexpression of miR-149-3p decreased the activity of *Wnt-1* 3′-UTR luciferase (*P* = 0.01, Figure 6B). To verify this result, we constructed another plasmid pmirGLO-Wnt1-Mut containing a mutation in the miR-149-3p binding sites. As expected, luciferase activity was increased if the seed region of *Wnt-1* was mutated (*P* = 0.02, Figure 6B). In addition, the protein level of Wnt-1 in MKN-1 cells decreased after pGL3-miR-149-3p transfection (Figure 6D). However, there was no increase in relative luciferase activity in HEK293T cells cotransfected with miR-149-3p inhibitor and pmirGLO-Wnt1-WT (Figure 6C), and only slightly changes were detected in Wnt-1 expression in MKN-1 cells transfected with miR-149-3p inhibitor (Figure 6D). This suggests that the interaction between the 3′-UTR of *Wnt-1* and low-levels of endogenous miR-149-3p was weak and therefore decreased miR-149-3p expression did not increase luciferase activity from the reporter vector. These results revealed that *Wnt-1* was a direct target gene of miR-149-3p.

## DISCUSSION

In the present study, we found that GRA ameliorated the inflammatory microenvironment by inhibiting the expression of inflammation-related genes. Additionally, the miRNA miR-149-3p was significantly upregulated by GRA in a dose- and time-dependent manner. We also confirmed that miR-149-3p played a tumor suppressor role by downregulating its direct target gene *Wnt-1*.

Chronic infection correlates with cancer in 15–20% of cases [10] and systemic inflammation is associated with poor prognosis in patients with advanced cancers [28]. Inflammation promotes GC development through the induction of the COX-2/PGE2 pathway in K19-C2mE transgenic (Tg) mice [8]. Here, our experiments with Tg mice showed that GRA inhibited the inflammatory response (Figure 1) and improved the inflammatory microenvironment in the gastric epithelial mucosa, where gastric cancer initiates and develops. Thiyagarajan and Chandrasekaran *et al.* showed that glycyrrhizin, from which GRA isextracted, did not exhibit anti-inflammatory activity inmurine macrophages (J774A.1) or human neutrophils (HL-60) [29, 30]. Therefore, we hypothesized that GRA acts primarily on epithelial cells.

GutGard is a novel, flavonoid-rich extract derived from *Glycyrrhiza glabra.* Although this extract showed a marked improvement of Nepean dyspepsia index [31], glycyrrhizin did not exhibit anti-inflammatory activity in the GutGard experiment. On the contrary, *Il-1β*, *Il-12b* and *Cox-2* were downregulated by GRA administration in our study (Figure 1E). Furthermore, the GutGard experiment only studied murine macrophages and human neutrophils and did not address the interaction of tumor cells with inflammatory microenvironments [29, 30]. On the other hand, the Tg mice used in our study developed gastric tumors spontaneously, emulating human gastric tumors morphologically and histochemically [8]. Therefore, the results of our experiments using Tg mice show GRA's anti-tumor effects *in vivo*.

MiRNAs contribute to cell proliferation and apoptosis and play important roles in tumor differentiation and metastasis. In our study, GRA administration upregulated miR-149-3p (Figure 4B). Our analyses showed that miR-149-3p functions as a tumor suppressor in GC (Figure 5). Indeed, miR-149-3p expression correlated negatively correlated with lymphonode metastasis (*P* = 0.02, Figure 4K), in agreement with other reports [32, 33]. Of note, pre-miR-149 transcripts exist in two different forms, 1) miR-149 transcribed from the 5′-end, and 2) miR-149-3p, which is transcribed from the 3′-end. Previous studies have shown that miR-149 inhibited cell proliferation, progression, migration, invasion and metastasis [32–34]. However, the tumor suppressor role of miR-149-3p was firstly identified in our study. Furthermore, we demonstrate that *Wnt-1* is a direct target gene of miR-149-3p (Figure 6). Other genes such as *PPM1F*, *GIT1*, *Rap1a*, *Rap1b, IL-6* and *EP2* have been previously identified as miR-149 targets [33–6].

Recently, miRNAs have attracted considerable attention, partly because multiple drugs have been shown to upregulate or activate them, thereby eliciting tumor-suppressive effects and yielding therapeutic benefits [9, 37–39]. In our study, GRA inhibited gastric tumorigenesis through the upregulation of tumor suppressor miR-149-3p. With the rapid development of miRNA profiling, an increasing number of miRNAs are being identified as therapeutic targets for various diseases. In addition to miR-149-3p, we found 13 other miRNAs that were upregulated and 22 that were downregulated in GC after GRA administration (Supplementary Table S1). For example, miR-328, a miRNA upregulated by GRA, inhibited cancer cell growth and impaired resistance to chemotherapeutic drugs by decreasing CD44 expression [40, 41]. Furthermore, miR-494, another miRNA upregulated by GRA, was shown to act as a tumor suppressor [42, 43]. In contrast, miR-223, a miRNA downregulated by GRA, was shown to function as an oncogene in human GC [44].

In conclusion, we propose that GRA inhibited the initiation and progression of gastric cancer by ameliorating the inflammatory microenvironment through inhibition of COX-2 expression, and by decreasing Wnt-1 expression through the upregulation of tumor suppressor miR-149-3p. Our results highlight GRA as a potential preventive and therapeutic agent against gastric cancer and warrant further studies to elucidate other molecular mechanisms involving GRA and additional miRNAs in GC and other types of cancer.

## MATERIALS AND METHODS

### Ethics statement

The use of the samples from animals and patients was approved by the Institutional Research Ethics Committee of Jilin University. Informed consent was obtained from all patients.

### Chemicals

18β-glycyrrhetinic acid (GRA) was purchased from Sigma-Aldrich (St. Louis, MO, USA). Stock solutions of GRA were dissolved in DMSO (vehicle) at 0.05% for *in vivo* studies and at 100 mM for *in vitro* studies as previously reported [18].

### Animal model and experimental protocol

K19-C2mE transgenic mice produced by the Oshima group [8] were maintained by breeding male Tg mice with female C57BL/6 N mice at the Animal Facility of Jilin University. Tg mice over-expressing *Cox-2* and *mPGES-1* in gastric epithelial cells were housed in plastic cages with free access to food and water. DNA was extracted from the tails of 4-week old mice and subjected to PCR as previously reported [9].

The transgenic male animals aged 6 weeks were divided into two groups randomly after genotyping (Figure 1A). The GRA-treated group (*n* = 40) was given to drink distilled water containing 0.05% GRA during the entire experimental period (52 weeks). The GRA solution was freshly prepared three times per week. Control mice (*n* = 40) were fed with the same diet and distilled water but without GRA.

### Tissue collection and histopathological assessment

The stomachs of Tg mice were resected, and pairs of tumor tissue and adjacent normal epithelial mucosa were preserved. The incidence, length, and width of tumors were calculated. All the samples were separated into two parts: one part was paraffin-embedded for hematoxylin-eosin (H&E) and immunohistochemical (IHC) staining, and the another part was flash-frozen in liquid nitrogen and preserved at −80°C until RNA extraction. For the grading of chronic active gastritis, a 4 point scale (G0, normal; G1, mild; G2, moderate; and G3, marked gastritis) was used according to criteria modified from the updated Sydney System [45]. Immunohistochemical detection of COX-2 (1:200, Santa Cruz, USA), β-catenin (1:200, Abcam, Cambridge, USA), Wnt-1 (1:200, Abcam), and H score calculation were performed as previously described [9].

### Patient samples and the cancer gnome atlas (TCGA) data analysis

For miRNA examination, 74 cases of gastric cancer and their paired control tissue (located > 3 cm away from the tumor) were obtained between September 2008 and January 2012 at First Hospital of Jilin University. The patient cohort consisted of 55 males and 19 females with a median age of 65.5 years (range, 39–90 years). The characteristics of the study patients are described in Table 2. For further verification, the miRNA data of 390 stomach adenocarcinoma and 42 paired normal gastric mucosa from the TCGA database (https://tcga-data.nci.nih.gov/tcga/tcgaHome2.jsp) were downloaded and analyzed. (These data had been obtained using Illumina HiSeq 2000 miRNA Sequencing System, Level 3).

**Table 2:** The relationship between clinical parameters and miR-149 expression (mean ± SD) in gastric adenocarcinoma

Clinical parameters	*N* (%)	Relative expression (−ΔCt)	*P*-value
Age (years)			
≧ 60	32 (43.2)	−4.96 ± 0.32	
< 60	42 (56.8)	−5.16 ± 0.26	0.64
Gender			
Male	55 (74.3)	−5.20 ± 0.25	
Female	19 (25.7)	−4.74 ± 0.30	0.33
Size (cm)			
≧ 5	39 (52.7)	−4.80 ± 0.22	
> 5	35 (47.3)	−5.39 ± 0.34	0.14
Differentiation grade			
Well to moderate	35 (47.3)	−5.39 ± 0.32	
Poor	39 (52.7)	−4.82 ± 0.25	0.15
T stage			
T1/2	15 (20.3)	−5.8 ± 0.43	
T3/4	59 (79.7)	−4.90 ± 0.23	0.08
N stage			
N0	18 (24.3)	−5.03 ± 0.52	
N1	20 (27.0)	−5.40 ± 0.61	0.02*
N2	16 (21.6)	−4.81 ± 0.26	0.12
N3	20 (27.0)	−5.33 ± 0.46	0.25
Distant metastasis			
M0	62 (83.8)	−4.97 ± 0.21	
M1	12 (16.2)	−4.97 ± 0.39	0.99
pTNM			
I/II	52 (70.3)	−5.38 ± 0.23	
III/IV	22 (29.7)	4.93 ± 0.30	0.24

### Cell culture

The human gastric tumor cell lines MKN-1 (well-differentiated), AGS (moderately-differentiated), MGC-803 (lowly-differentiated), BGC-823 (undifferentiated), as well as the immortal gastric epithelia cell lines GES-1 and HEK-293T were cultured in a 5% CO_2_ incubator at 37°C. RPMI 1640 and DMEM media (Hyclone, Logan, Utah, USA) containing 10% heat-inactivated fetal bovine serum (FBS, Gibco, New York, USA), penicillin (100 U/ml) and streptomycin (100 U/ml) (Hyclone) were used for cell culture.

### Cell transfection

The hsa-miR-149-3p was cloned and constructed into a pGL3 vector to create a pGL3-miR-149-3p plasmid, and a blank plasmid was used as the control (miR-CK). In addition, the human miR-149-3p inhibitor and a negative control (miR-NC) were designed and provided by Ribobio (Guangzhou, Guangdong, China). Eight μg pGL3-miR-149-3p/miR-CK or 200 nM miR-149-3p inhibitor/miR-NC were transfected using Lipofectamine 2000 (1:2, Invitrogen, Carlsbad, USA).

### Cell viability assay

The anti-proliferative effects of GRA were determined using MTT assay as previously reported [46]. The gastric tumor cell lines BGC-823 and MKN-1 were seeded in 96-well plates and treated with serial dilutions of GRA (0, 50, 100, 150, 200 μM) (*n* = 10) for 4 h, 24 h and 48 h. The immortal gastric epithelia cell line GES-1 was used as a control. Cell viability was assessed using an MTT Kit (Promega, Madison, USA) and absorbance was measured at 490 nm using a micro plate reader (Thermo, MA, USA).

### Colony formation assay

The ability of gastric tumor cells to divide unlimitedly after transfection with miR-149-3p or miR-149-3p inhibitor was tested using colony formation assay as previously described [47]. The gastric tumor cell lines MKN-1 and BGC-823 were planted in 24-well plates with 4 × 10^5^ cells per well, and transfected with 8 μg miR-149-3p/miR-CK or 200 nM miR-149-3p inhibitor/miR-NC. After 24 h, the cells were trypsinized and reseeded in 6-cm plates with 1 × 10^3^ cells per well, and cultured for 10 days. The colonies were stained with 1% crystal violet for 15 min after fixation with 4% neutral formaldehyde for 15 min. All experiments were performed in triplicate.

### Flow cytometry analysis of apoptosis

MKN-1 and BGC-823 cells were seeded in 6-cm plates, and treated with serial dilutions of GRA (0, 100, and 150 μM) (*n* = 3) for 24 h. In addition, MKN-1 and BGC-823 cells were seeded in 24-well plates, and were transfected with 8 μg miR-149-3p/miR-CK or 200 nM miR-149-3p inhibitor/miR-NC for 24 h. Apoptotic and necrotic cells were evaluated by Annexin V (AV) binding and 7-AAD or PI uptake (BD Bioscience, Bedford, MA, USA). All the samples were analyzed by flow cytometry (BD Bioscience).

### Assessment of cell cycle

After exposure to GRA (0, 100, and 150 μM) or transfection with 8 μg miR-149-3p/miR-CK or 200 nM miR-149-3p inhibitor/miR-NC for 24 h (*n* = 3), MKN-1 and BGC-823 cells were collected and stained with PI in the dark. The cell cycle was analyzed by flow cytometry (BD Bioscience).

### Total RNA rich of miRNA isolation

Total RNA rich of miRNA isolation in Tg mouse gastric tumors, MKN-1 and BGC-823 cells with and without GRA treatment, as well as in tumors from gastric cancer patients, were extracted using the miRNeasy Kits (Qiagen, Hilden, Germany), and reversed transcribed using miScript II RT Kit (Qiagen) for miRNA quantification, or reversed transcribed using cDNA synthesis kits (Roche, Basal, Switzerland) for mRNA quantification according to the manufacturer's protocol.

### MicroRNA microarray

Total RNA rich of miRNA isolation in the GRA-treated (*n* = 5) and control (*n* = 5) groups was extracted and analyzed by Affymetrix Mouse miRNA array (v.3.0). Each microarray chip was hybridized with a single sample labeled with either Cy3 or Cy5. The raw data were deposited at Shanghai Biotechnology Corporation (Shanghai, China). Background subtraction and normalization were performed using Expression Console software. The miRNAs that exhibited a significant difference in levels (≥ 2-fold, *P* < 0.05) were selected.

### miRNA and mRNA quantification

miR-149-3p was quantified using specific mmu-miR-149-3p primer (Qiagen) in Tg mice, and hsa-miR-149-3p primer (Qiagen) in gastric tumor cells and patient samples by qRT-PCR (Roche LC480 II). RNU6 was used as the internal reference, and the mRNA levels of *Cox-2*, *mPGES-1*, *Il-1b*, and *Il-12b* normalized to *GAPDH* were also analyzed. The 2^−ΔΔCt^ method was adopted.

### Dual-luciferase reporter assay

Hsa-miR-149-3p was cloned and constructed into pGL3 vector to create pGL3-miR-149-3p. *Wnt-1* wildtype and mutant 3′-UTR were cloned and constructed independently into pmirGLO vector to create pmirGLO-Wnt-1-WT and pmirGLO-Wnt-1-Mut, respectively. All the vectors were provided by Shanghai GeneChem Corporation. HEK-293T cells were cotransfected with pGL3-miR-149-3p or miR-149-3p inhibitor and pmirGLO-Wnt-1-WT/Mut using Lipofectamine2000 (Invitrogen) in a 24-well plate. Twenty four hours after transfection, luciferase assay was performed using a dual-luciferase reporter assay kit (Promega, Wisconsin, USA).

### Western blot analysis

The total protein of gastric tumor cells was extracted using a mammalian protein extraction kit (Kangwei, China), and the concentration of various proteins was measured using a BCA kit (Kangwei). The levels of COX-2 (1:1,000, Santa Cruz), Wnt-1, β-catenin, PTEN, Akt, p-Akt (Ser 473), c-Raf, p-c-Raf (Ser 338), p44/42 MAPK (Erk1/2) and p-p44/42 MAPK (Erk1/2) (Thr202/Tyr204) and GAPDH (1:1,000, Abcam) were measured with ECL reagents (Thermo Fisher Scientific) using Molecular Imager ChemiDox XRS+ imaging system (Biorad, California, USA).

### Statistics

All analyses were performed using SPSS version 10.0 (SPSS Inc., USA) or GraphPad Prism 5.0. Data were evaluated using one-way ANOVA. A value of *P* < 0.05 was considered statistically significant while *P* < 0.01 and *P* < 0.001 were highly significant and very highly significant, respectively.

## SUPPLEMENTARY MATERIALS TABLE



## References

[1] Torre LA, Bray F, Siegel RL, Ferlay J, Lortet-Tieulent J, Jemal A (2015). Global cancer statistics, 2012. CA Cancer J Clin.

[2] Chen WQ, Zheng RS, Baade PD, Zhang SW, Zeng HM, Bray F, Jemal A, Yu XQ, He J (2016). Cancer statistics in China, 2015. CA Cancer J Clin.

[3] Yang JL, Zhu LL, Wu ZY, Wang YP (2013). Chinese herbal medicines for induction of remission in advanced or late gastric cancer. The Cochrane database of systematic reviews.

[4] Li K, Li J (2016). Current Molecular Targeted Therapy in Advanced Gastric Cancer: A Comprehensive Review of Therapeutic Mechanism, Clinical Trials, and Practical Application. Gastroenterology research and practice.

[5] Smyth EC, Cunningham D (2016). Encouraging results for PD-1 inhibition in gastric cancer. The Lancet Oncology.

[6] Grivennikov SI, Greten FR, Karin M (2010). Immunity, inflammation, and cancer. Cell.

[7] Correa P (1988). A human model of gastric carcinogenesis. Cancer research.

[8] Oshima H, Oshima M, Inaba K, Taketo MM (2004). Hyperplastic gastric tumors induced by activated macrophages in COX-2/mPGES-1 transgenic mice. The EMBO journal.

[9] Cao DH, Jiang J, Tsukamoto T, Liu RM, Ma L, Jia ZF, Kong F, Oshima M, Cao XY (2015). Canolol Inhibits Gastric Tumors Initiation and Progression through COX-2/PGE2 Pathway in K19-C2mE Transgenic Mice. PLoS One.

[10] Fichtner-Feigl S, Kesselring R, Strober W (2015). Chronic inflammation and the development of malignancy in the GI tract. Trends in immunology.

[11] Oshima H, Matsunaga A, Fujimura T, Tsukamoto T, Taketo MM, Oshima M (2006). Carcinogenesis in mouse stomach by simultaneous activation of the Wnt signaling and prostaglandin E2 pathway. Gastroenterology.

[12] Zhang B, Yang Y, Shi X, Liao W, Chen M, Cheng AS, Yan H, Fang C, Zhang S, Xu G, Shen S, Huang S, Chen G (2015). Proton pump inhibitor pantoprazole abrogates adriamycin-resistant gastric cancer cell invasiveness via suppression of Akt/GSK-beta/beta-catenin signaling and epithelial-mesenchymal transition. Cancer letters.

[13] Mao J, Fan S, Ma W, Fan P, Wang B, Zhang J, Wang H, Tang B, Zhang Q, Yu X, Wang L, Song B, Li L (2014). Roles of Wnt/beta-catenin signaling in the gastric cancer stem cells proliferation and salinomycin treatment. Cell death & disease.

[14] Li Y, Sun WG, Liu HK, Qi GY, Wang Q, Sun XR, Chen BQ, Liu JR (2011). gamma-Tocotrienol inhibits angiogenesis of human umbilical vein endothelial cell induced by cancer cell. The Journal of nutritional biochemistry.

[15] Kim YM, Kim IH, Nam TJ (2013). Capsosiphon fulvescens glycoprotein inhibits AGS gastric cancer cell proliferation by downregulating Wnt-1 signaling. International journal of oncology.

[16] Shen WD, Zou XP, Chen M, Liu PF, Shen YH, Huang SL, Guo HM, Zhang LL (2011). Effects of diphyllin as a novel V-ATPase inhibitor on gastric adenocarcinoma. European journal of pharmacology.

[17] Park CH, Hahm ER, Lee JH, Jung KC, Yang CH (2005). Inhibition of beta-catenin-mediated transactivation by flavanone in AGS gastric cancer cells. Biochemical and biophysical research communications.

[18] Cao DH, Jiang J, You LL, Jia ZF, Tsukamoto T, Cai HK, Wang SD, Hou Z, Suo YE, Cao XY (2016). The protective effects of 18β-glycyrrhetinic acid on Helicobacter pylori-Iinfected gastric mucosa in Mongolian Gerbils. Biomed Research International.

[19] Wang XF, Zhou QM, Lu YY, Zhang H, Huang S, Su SB (2015). Glycyrrhetinic acid potently suppresses breast cancer invasion and metastasis by impairing the p38 MAPK-AP1 signaling axis. Expert Opin Ther Targets.

[20] Kong SZ, Chen HM, Yu XT, Zhang X, Feng XX, Kang XH, Li WJ, Huang N, Luo H, Su ZR (2015). The protective effect of 18beta-Glycyrrhetinic acid against UV irradiation induced photoaging in mice. Experimental gerontology.

[21] Zhu LC, Yang X, Tan J, Wang BC, Zhang X (2014). A validated high performance liquid chromatograph-photodiode array method for simultaneous determination of 10 bioactive components in compound hongdoushan capsule. Pharmacognosy magazine.

[22] Tang ZH, Zhang LL, Li T, Lu JH, Ma DL, Leung CH, Chen XP, Jiang HL, Wang YT, Lu JJ (2015). Glycyrrhetinic acid induces cytoprotective autophagy via the inositol-requiring enzyme 1alpha-c-Jun N-terminal kinase cascade in non-small cell lung cancer cells. Oncotarget.

[23] Lin DJ, Zhong W, Li J, Zhang B, Song G, Hu TH (2014). Involvement of BID translocation in glycyrrhetinic acid and 11-deoxy glycyrrhetinic acid-induced attenuation of gastric cancer growth. Nutr Cancer.

[24] Wu CH, Chen AZ, Yen GC (2015). Protective effects of glycyrrhizic acid and 18beta-glycyrrhetinic acid against cisplatin-Induced Nephrotoxicity in BALB/c Mice. J Agric Food Chem.

[25] Chiurillo MA (2015). Role of the Wnt/beta-catenin pathway in gastric cancer: An in-depth literature review. World journal of experimental medicine.

[26] Kong D, Piao YS, Yamashita S, Oshima H, Oguma K, Fushida S, Fujimura T, Minamoto T, Seno H, Yamada Y, Satou K, Ushijima T, Ishikawa TO (2012). Inflammation-induced repression of tumor suppressor miR-7 in gastric tumor cells. Oncogene.

[27] Zheng HY, Zhang FX, Lin XJ, Huang CM, Zhang YQ, Li Y, Lin JY, Chen WN, Lin X (2016). MicroRNA-1225-5p inhibits proliferation and metastasis of gastric carcinoma through repressing insulin receptor substrate-1 and activation of beta-catenin signaling. Oncotarget.

[28] Hwang JE, Kim HN, Kim DE, Choi HJ, Jung SH, Shim HJ, Bae WK, Hwang EC, Cho SH, Chung IJ (2011). Prognostic significance of a systemic inflammatory response in patients receiving first-line palliative chemotherapy for recurred or metastatic gastric cancer. BMC cancer.

[29] Thiyagarajan P, Chandrasekaran CV, Deepak HB, Agarwal A (2011). Modulation of lipopolysaccharide-induced pro-inflammatory mediators by an extract of Glycyrrhiza glabra and its phytoconstituents. Inflammopharmacology.

[30] Chandrasekaran CV, Deepak HB, Thiyagarajan P, Kathiresan S, Sangli GK, Deepak M, Agarwal A (2011). Dual inhibitory effect of Glycyrrhiza glabra (GutGard) on COX, LOX products. Phytomedicine.

[31] Raveendra KR, Jayachandra, Srinivasa V, Sushma KR, Allan JJ, Goudar KS, Shivaprasad HN, Venkateshwarlu K, Geetharani P, Sushma G, Agarwal A (2012). An Extract of Glycyrrhiza glabra (GutGard) Alleviates Symptoms of Functional Dyspepsia: A Randomized, Double-Blind, Placebo-Controlled Study. Evidence-Based Complementary and Alternative Medicine.

[32] Wang Y, Zheng XS, Zhang ZY, Zhou JF, Zhao GH, Yang JJ, Xia LM, Wang R, Cai XQ, Hu H, Zhu CL, Nie YZ, Wu KC (2012). MicroRNA-149 inhibits proliferation and cell cycle progression through the targeting of ZBTB2 in human gastric cancer. PLoS One.

[33] Luo G, Chao YL, Tang B, Li BS, Xiao YF, Xie R, Wang SM, Wu YY, Dong H, Liu XD, Yang SM (2015). miR-149 represses metastasis of hepatocellular carcinoma by targeting actin-regulatory proteins PPM1F. Oncotarget.

[34] Bischoff A, Huck B, Keller B, Strotbek M, Schmid S, Boerries M, Busch H, Muller D, Olayioye MA (2014). miR149 functions as a tumor suppressor by controlling breast epithelial cell migration and invasion. Cancer research.

[35] Chan SH, Huang WC, Chang JW, Chang KJ, Kuo WH, Wang MY, Lin KY, Uen YH, Hou MF, Lin CM, Jang TH, Tu CW, Lee YR (2014). MicroRNA-149 targets GIT1 to suppress integrin signaling and breast cancer metastasis. Oncogene.

[36] Li P, Shan JX, Chen XH, Zhang D, Su LP, Huang XY, Yu BQ, Zhi QM, Li CL, Wang YQ, Tomei S, Cai Q, Ji J (2015). Epigenetic silencing of microRNA-149 in cancer-associated fibroblasts mediates prostaglandin E2/interleukin-6 signaling in the tumor microenvironment. Cell research.

[37] Saito Y, Suzuki H, Imaeda H, Matsuzaki J, Hirata K, Tsugawa H, Hibino S, Kanai Y, Saito H, Hibi T (2013). The tumor suppressor microRNA-29c is downregulated and restored by celecoxib in human gastric cancer cells. International journal of cancer.

[38] Ru Q, Tian X, Pi MS, Chen L, Yue K, Xiong Q, Ma BM, Li CY (2015). Voltagegated K+ channel blocker quinidine inhibits proliferation and induces apoptosis by regulating expression of microRNAs in human glioma U87MG cells. International journal of oncology.

[39] Tang HL, Kong YN, Guo JL, Tang Y, Xie XH, Yang L, Su Q, Xie XM (2013). Diallyl disulfide suppresses proliferation and induces apoptosis in human gastric cancer through Wnt-1 signaling pathway by up-regulation of miR-200b and miR-22. Cancer letters.

[40] Ishimoto T, Izumi D, Watanabe M, Yoshida N, Hidaka K, Miyake K, Sugihara H, Sawayama H, Imamura Y, Iwatsuki M, Iwagami S, Baba Y, Horlad H (2015). Chronic inflammation with Helicobacter pylori infection is implicated in CD44 overexpression through miR-328 suppression in the gastric mucosa. J Gastroenterol.

[41] Ishimoto T, Sugihara H, Watanabe M, Sawayama H, Iwatsuki M, Baba Y, Okabe H, Hidaka K, Yokoyama N, Miyake K, Yoshikawa M, Nagano O, Komohara Y (2014). Macrophage-derived reactive oxygen species suppress miR-328 targeting CD44 in cancer cells and promote redox adaptation. Carcinogenesis.

[42] He WL, Li YH, Chen XL, Lu LY, Tang B, Wang ZX, Pan YB, Cai SR, He YL, Ke ZF (2014). miR-494 acts as an anti-oncogene in gastric carcinoma by targeting c-myc. Journal of gastroenterology and hepatology.

[43] Zhou RP, Chen G, Shen ZL, Pan LQ (2014). Cinobufacin suppresses cell proliferation via miR-494 in BGC- 823 gastric cancer cells. Asian Pacific journal of cancer prevention.

[44] Zhou XY, Jin WJ, Jia HY, Yan J, Zhang GX (2015). MiR-223 promotes the cisplatin resistance of human gastric cancer cells via regulating cell cycle by targeting FBXW7. J Exp Clin Cancer Res.

[45] Cao XY, Tsukamoto T, Nozaki K, Tanaka H, Cao LY, Toyoda T, Takasu S, Ban H, Kumagai T, Tatematsu M (2007). Severity of gastritis determines glandular stomach carcinogenesis in Helicobacter pylori-infected Mongolian gerbils. Cancer Sci.

[46] Jiang J, Cao DH, Tsukamoto T, Wang GQ, Jia ZF, Suo J, Cao XY (2013). Anticancer effects of 4-vinyl-2, 6-dimethoxyphenol (canolol) against SGC-7901 human gastric carcinoma cells. Oncology letters.

[47] Franken NA, Rodermond HM, Stap J, Haveman J, van Bree C (2006). Clonogenic assay of cells *in vitro*. Nature protocols.

