# Supplementary material for: 18β-glycyrrhetinic acid suppresses gastric cancer by activation of miR-149-3p-Wnt-1 signaling
Source: Oncotarget. 2016 Oct 4;7(44):71960–73. doi: 10.18632/oncotarget.12443 (PMC5342136; doi:10.18632/oncotarget.12443)
Supplement: Supplementary file 1 [file oncotarget-07-71960-s001.pdf]

18β-glycyrrhetinic acid suppresses gastric cancer by activation of miR-149-3p-Wnt-1 signaling

Supplementary Materials

Supplementary Table S1: Differential expression of miRNAs in GRA-treated Tg gastric cancer

Regulation	miRNA	Fold change	*P* value
Upregulated (*n* = 16)	miR-149-3p	3.84	0.01
	miR-3472	3.39	0.01
	miR-3077	3.35	0.02
	miR-714	3.34	0.04
	miR-5135	3.30	0.02
	miR-720	3.24	0.02
	miR-3081	3.16	0.02
	miR-466f-3	2.80	0.02
	miR-494	2.67	0.02
	miR-3474	2.60	0.00
	miR-3960	2.59	0.01
	miR-2861	2.57	0.02
	miR-1982	2.56	0.01
	miR-328	2.20	0.05
	miR-1892	2.08	0.04
	miR-5109	2.02	0.01
Downregulated (*n* = 22)	miR-125b-1-3p	0.48	0.02
	miR-486	0.48	0.03
	miR-34c	0.47	0.04
	miR-212-3p	0.46	0.02
	miR-3102	0.44	0.04
	miR-872	0.44	0.04
	miR-301a	0.43	0.03
	miR-674	0.42	0.01
	miR-3060	0.42	0.01
	miR-93	0.41	0.01
	miR-501-5p	0.41	0.01
	miR-30a	0.41	0.00
	miR-34c	0.39	0.04
	miR-7a-1	0.38	0.05
	miR-1843-5p	0.38	0.02
	miR-140	0.37	0.00
	miR-326	0.37	0.03
	miR-128	0.34	0.00
	miR-215	0.32	0.00
	miR-223	0.31	0.03
	miR-212-5p	0.27	0.00
	miR-153	0.26	0.02
